# Systematic comparison of variant calling pipelines of target genome sequencing cross multiple next-generation sequencers

**DOI:** 10.3389/fgene.2023.1293974

**Published:** 2024-01-04

**Authors:** Baosheng Feng, Juan Lai, Xue Fan, Yongfeng Liu, Miao Wang, Ping Wu, Zhiliang Zhou, Qin Yan, Lei Sun

**Affiliations:** ^1^ GeneMind Biosciences Company Limited, Shenzhen, China; ^2^ Clinical Research Institute, Shanghai General Hospital, Shanghai Jiao Tong University School of Medicine, Shanghai, China

**Keywords:** HD832, TSO500, NGS, target genome sequencing, SNVer, Varscan 2

## Abstract

Targeted genomic sequencing (TS) greatly benefits precision oncology by rapidly detecting genetic variations with better accuracy and sensitivity owing to its high sequencing depth. Multiple sequencing platforms and variant calling tools are available for TS, making it excruciating for researchers to choose. Therefore, benchmarking study across different platforms and pipelines available for TS is imperative. In this study, we performed a TS of Reference OncoSpan FFPE (HD832) sample enriched by TSO500 panel using four commercially available sequencers, and analyzed the output 50 datasets using five commonly-used bioinformatics pipelines. We systematically investigated the sequencing quality and variant detection sensitivity, expecting to provide optimal recommendations for future research. Four sequencing platforms returned highly concordant results in terms of base quality (Q20 > 94%), sequencing coverage (>97%) and depth (>2000×). Benchmarking revealed good concordance of variant calling across different platforms and pipelines, among which, FASTASeq 300 platform showed the highest sensitivity (100%) and precision (100%) in high-confidence variants calling when analyzed by SNVer and VarScan 2 algorithms. Furthermore, this sequencer demonstrated the shortest sequencing time (∼21 h) at the sequencing mode PE150. Through the intersection of 50 datasets generated in this study, we recommended a novel set of variant genes outside the truth set published by HD832, expecting to replenish HD832 for future research on tumor variant diagnosis. Besides, we applied these five tools to another panel (TargetSeq One) for Twist cfDNA Pan-cancer Reference Standard, comprehensive consideration of SNP and InDel sensitivity, SNVer and VarScan 2 performed best among them. Furthermore, SNVer and VarScan 2 also performed best for six cancer cell lines samples regarding SNP and InDel sensitivity. Considering the dissimilarity of variant calls across different pipelines for datasets from the same platform, we recommended an integration of multiple tools to improve variant calling sensitivity and accuracy for the cancer genome. Illumina and GeneMind technologies can be used independently or together by public health laboratories performing tumor TS. SNVer and VarScan 2 perform better regarding variant detection sensitivity for three typical tumor samples. Our study provides a standardized target sequencing resource to benchmark new bioinformatics protocols and sequencing platforms.

## 1 Introduction

Targeted genomic sequencing (TS) is an effective next-generation sequencing (NGS) method that focuses on a panel of genes or targets implicated in the pathogenesis or clinical relevance. It greatly benefits precision oncology by rapidly detecting genetic variations, providing a resource for clinicians to help them interpret genetic profiles, and implement personalized anticancer recommendations. TS shows better accuracy and sensitivity in identifying targeted variations owing to greater sequencing depth at the same sequencing cost and data burden when compared with whole-genome sequencing (WGS) or whole-exome sequencing (WES) (Bewicke-Copley et al., 2019). Thus, it allows for identifying mutations presenting low variant allele frequencies (VAFs) with high confidence, especially for low-quality and fragmented clinical DNA samples. Clinical targeted sequencing has revolutionized tumor surveillance and diagnosis and facilitated the development of precision oncology (Nagahashi et al., 2019; Tan et al., 2020a).

TruSight™ Oncology 500 (TSO500, Illumina) is a comprehensive target-sequencing panel covering more than eight cancer types and 523 cancer-related genes (1.94 Mb) to identify relevant genomic variants and signatures in a single assay (Zhao et al., 2020). It uses a hybridization capture-based target-enrichment strategy to detect diverse variants with high specificity and sensitivity, including single nucleotide polymorphisms (SNPs), InDels, copy number variations, splice variants and gene fusions, especially for mutants with low VAFs via tactfully suppressing technical noise and excluding germline variants (Zhao et al., 2020; Conroy et al., 2021). Notably, TSO500 is also robust for tumor mutational burden (TMB) detection with a relatively high concordance compared with WES in different solid tumor samples (Wei et al., 2022).

Currently, TSO500 panel has been benchmarked and verified against orthogonal validated NGS assays (such as WGS, WES) using diverse clinical tissue specimens across different tumor types (Pestinger et al., 2020; Sahajpal et al., 2020; Conroy et al., 2021; Ramos-Paradas et al., 2021; Wei et al., 2022). The high concordance with the reference methods demonstrates that the TSO500 assay is reliable for accurately detecting gene alterations to support the precision therapeutics in oncology. While NGS has revolutionized the understanding of disease diagnosis and prediction, the magnitude of sequencing data points toward the potential challenges for advancing large-scale executions-the appropriate platform and bioinformatics pipeline capable of handling these data efficiently in a timely and accurate manner. In this regard, the need for benchmarking across different sequencing platforms and bioinformatics analysis tools for TS supporting precision diagnostics is imperative. Regrettably, the systematical benchmarking studies of the TSO500 panel on different sequencing platforms and bioinformatics pipelines are still blank. In addition, the Twist Pan-cancer Reference Standard is a high-quality, standardized control for researching and developing NGS-based liquid biopsy assays, primarily used to track the quality of an NGS assay workflow and assess the fidelity of the assay process (Cherry, 2022). Besides our study has also included the published panel sequencing data of six ovarian and breast cancer cell lines (Shi et al., 2022).

In this study, we comprehensively compared six commercially available sequencers (NA: NovaSeq 6000, NS: NextSeq 550, MGI: MGISEQ-2000, GL: GenoLab M, SF: SURFSeq 5000, and FS: FASTASeq 300) and five commonly-used bioinformatics pipelines for tumor variants detection. These pipelines were highly cited or could detect low-frequency variation by adjusting the appropriate parameters. HaplotypeCaller (HC) (McKenna et al., 2010) and Mutect2 (Benjamin et al., 2019) were GATK-related tools. SiNVICT (Kockan et al., 2017) and SNVer (Wei et al., 2011) with short release time and long release time, respectively, and VarScan 2 (Koboldt et al., 2012) had been well recognized with more than 4,500 citations (Supplementary Table S1). The consistency and dissimilarity of different platforms and pipelines were evaluated in terms of SNP, InDel and TMB calling. By comparing with the truth set, we expected to provide objective insights into platforms and variant callers to achieve higher sensitivity, which can be crucial in cancer research and personalized medicine.

## 2 Materials and methods

### 2.1 Sample

The DNA sample used in this study is OncoSpan FFPE (Catalog ID: HD832, Horizon Discovery, United States), a well-characterized, cell line-derived Reference Standard containing 386 variants in 152 cancer genes (https://horizondiscovery.com/en/reference-standards/products/oncospan-gdna). In this study, the mutation cohort captured by the TSO500 panel theoretically includes 212 variants (194 SNPs and 18 InDels), with 24 confirmed by droplet digital PCR (ddPCR). This dataset was designed as a truth set for following benchmarking analyses. The VAF varies between 1% and 100%. The cfDNA sample used is Twist Pan-cancer Reference Standard (MineBio Life Sciences Ltd., Beijing, China), covering 458 individual mutations with 132 clinically actionable variants across 84 genes associated with cancer.

### 2.2 DNA extraction and quality assessment

WuXi Nextcode LTD. performed DNA isolation from OncoSpan scroll using the SEQPLUS FFPE DNA Isolation Kit following the manufacturer’s protocol. The DNA quality with OD 260/280 value between 1.7 and 2.2 was qualified by a Nanodrop spectrophotometer. The integrity and concentration of the extracted DNA were confirmed by agarose electrophoresis and Qubit dsDNA HS Assay (Thermo Fisher Scientific). About 40 ng DNA sample was used for library preparation.

### 2.3 Library preparation and target enrichment

The extracted DNA passing quality control (QC) was sheared on the Covaris E220 evolution (Covaris Ltd., United States) to form 90–250 bp dsDNA fragments. The size of the fragments was chosen *via* Tapestation 2200 (Agilent, Cheshire, UK) after shearing. Then, the sequencing libraries were prepared and enriched using the hybrid capture-based TSO500 library preparation kit (#20028213, TruSight Oncology 500 DNA Kit, Illumina, San Diego, CA, United States) following the manufacturer’s instruction.

In brief, the sheared DNA was treated with end repair and A-tailing reagents to convert the 3′and 5′overhangs into blunt ends. UMI1 adapters containing unique molecular indexes were ligated to identify the unique sequence. After cleaning up the excessive ligation reagents and unligated adapters, the library fragments were amplified using primers that added index sequences for sample multiplexing. Next, the libraries were enriched through two rounds of hybridization capture. A pool of oligos specific to 523 genes (TSO500 panel) were used to hybridize to the DNA libraries, which were later captured with SMB (Streptavidin Magnetic Beads)-conjugated biotin probes. Subsequently, the enriched libraries were amplified, quantified with the Qubit dsDNA HS Assay Kit (#Q32854, Invitrogen, United States), and bead-based normalized for sequencing. The Twist cfDNA Pan-cancer Reference Standard was enriched by TargetSeq One kit (iGeneTech, Inc, Beijing, China.) referred to a previous study (Nie et al., 2021).

### 2.4 Sequencing, data preparation and quality control

Before sequencing, the size distribution of the sequencing library HD832 was characterized using Agilent Tapestation 4,200. WuXi Nextcode LTD. carried out HD832 library preparation and sequencing on Illumina NS and NA platforms in Shanghai. Meanwhile, sequencing on the FS and GL platforms was performed at the lab of GeneMind LTD. in Shenzhen. Besides, Twist cfDNA was sequenced by SF, NA, and MGI at the lab of GeneMind LTD. The sequencing mode is PE150 bp.

Raw data from FFPE, cfDNA, and cancer cell samples was filtered by fastp software to trim sequencing adapters, and low-quality reads with default parameters. The reads were filtered out if the proportion of low-quality bases (<Q20) was higher than 15%. The sequencing reads were mapped using to the human reference genome (hg19) with default parameters via Burrows-Wheeler Aligner (BWA v0.7.17-r1188)-Maximal Exact Match (MEM) algorithm. Hereafter, the samtools and GATK “Mark Duplicates (Picard)” module were used to implement the following processes, including indexing, sorting, and duplicates removal in BAM files. The deduplicated BAM files were subjected to different pipelines for variant calling. Quality metrics were generated from these BAM files by fastQC and bamdst tools.

### 2.5 Benchmarking analyses of variant calling among five variant callers

The tumor mutations were called with five popular pipelines: HC, Mutect2, SiNVICT, SNVer, and VarScan 2 with default parameters. To reduce false-positive calls, eligible mutations include only coding variants with VAF ≥5%, coverage ≥8 reads, and allele read depth ≥3×. Next, the variants were annotated for impact prediction with snpEFF software. Broadening to the whole panel range, we calculated the somatic mutations through aligning against four common population databases: Exome Aggregation Consortium, 1000 Genomes Project, Database of Short Genetic Variations, and Exome Sequencing Project v. 6500 to remove the common germline variants (VAF >0.1%). Then, variants were labeled germline if they were regarded as a benign or likely benign variant in either the Human Gene Mutation Database or ClinVar. In addition to these common frequency databases, the depth of somatic variants required DP ≥ 100, AD ≥ 8 for SNVs, and AD ≥ 5 for InDels (Tang et al., 2020b). TMB was computed as a ratio between the numbers of somatic mutations with the target region size of the panel (Merino et al., 2020). We further implemented the correlation analysis by comparing relative variant allele frequency (r-VAF), defined as the ratio of detected value to the theoretical value of the truth set, to excavate the concordance of 50 datasets.

### 2.6 Statistical analysis

All datasets were analyzed using the R statistics package (v4.1.2; R: The R-project for statistical computing). The similarity of datasets was calculated using pearson’s correlation coefficient. Precision, recall (sensitivity), and F-score were calculated based on the true sets of 212 mutations in the TSO500 panel, 442 mutations in TargetSeq One, and true mutations in ovarian and breast cancer cell lines referred to Pan et al., 2022. The following formulas were used.

$$\text{Precision}=\text{TP}/\left(\text{TP}+\text{FP}\right).$$

$$\text{Sensitivity}=\text{TP}/\left(\text{TP}+\text{FN}\right).$$

$$F-\text{score}=2*\text{Sensitivity}*\text{Precision}/\left(\text{Sensitivity}+\text{Precision}\right).$$

TP:true positive,FN:false negative,FP:false positive.

## 3 Result

### 3.1 Sequencing data summary

We aliquot the same DNA sequencing library (HD832 captured by TSO500 panel) to each platform to avoid the possibility of inconsistency caused by library construction differences. After filtering, aligning and deduplicating, 10 datasets (FS 3, GL 3, NA 3, and NS 1) were subjected to five analysis pipelines for variant calling: HC, Mutect2, SiNVICT, SNVer and VarScan 2 (Supplementary Figure S1). FASTQ and BAM quality statistics were calculated for the final 50 datasets, as shown in Supplementary Table S2. Benchmarking showed that results from GeneMind platforms (FS and GL) are compatible with those using Illumina platforms (NA and NS) in terms of the data yield and data quality. All sequencers presented comparable high base quality (over Q20) base percentages, with an average of 96.49% (FS), 97.40% (NA), 97.01% (GL) and 94.05% (NS). Each dataset from four sequencing platforms has a trustworthy average depth, higher than 2000×. The short target sequence length and great sequencing depth contributed to the high duplication. To be compatible with relatively low coverage regions, we downsampled data with a depth ≥4×, ≥10×, ≥30×, and ≥100×, and the coverage at different levels for all datasets was over 97%. The average depth for each gene across multiple sequencers ranged from 31.56 to 4,823.42 × (Supplementary Table S3). Overall, the datasets from four sequencing platforms returned highly concordant quality, achieving the requirement of the high depth of panel sequencing with approving uniformity.

### 3.2 High-confidence variants calling spanning four platforms and five pipelines

**FIGURE 1:**
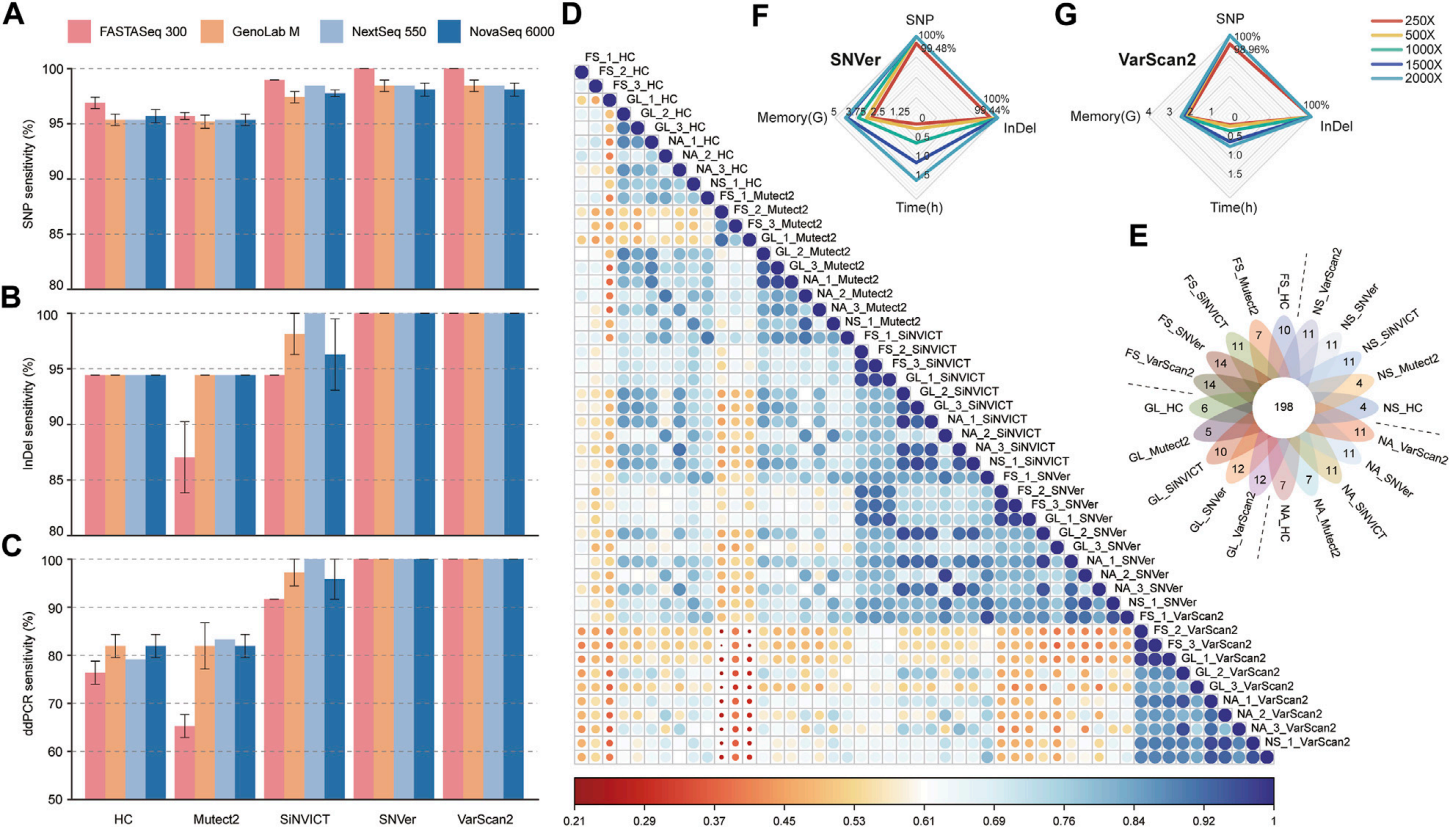
Benchmarking analyses of variants calling performances compared with truth set. **(A, B)** The sensitivity of SNP (a) and InDel (b) calls generated by five pipelines *versus* that from four platforms. **(C)** Comparison of SNP&InDel calls with variants validated by ddPCR. **(D)** Pearson’s correlation heatmap of all 50 datasets based on the relative VAF. **(E)** Venn diagram showed the number of variants mapped to the target region spanning four platforms and five pipelines. The digital of petals represents the number of specific mutations detected in the corresponding dataset, and the flower center represents the number of common mutations detected in all datasets. **(F, G)** The radar charts compared the dissimilarity at different sequencing depths (FS platform) in terms of variant calling time, memory usage, SNP and InDel detection sensitivity using SNVer (f) and VarScan2 (g) tools. The fastq files of different depths were downsampled from different runs of the FS sequencer. FS, FASTASeq 300; NA, NovaSeq 6000; NS, NextSeq 550; GL, GenoLab M; HC, GATK_HaplotypeCaller; Mutect2, GATK_Mutect2.

Subsequently, we investigated the SNP&InDel calling rate of four platforms with five popular analysis algorithms at 2000× depth by comparing with the truth set of the HD832 sample captured by the TSO500 panel. As shown in Figures 1A, B, four platforms presented nearly concordant results in terms of SNP&InDel sensitivity under the same pipeline, albeit with some minor differences in the InDel calling using SiNVICT and Mutect2 callers. Encouragingly, the FS platform showed perfect SNP&InDel calling (100%) using SNVer and VarScan2 pipelines. For F-score and precision, SNVer and VarScan2 pipelines also performed best on SNP&InDel calling (Supplementary Table S4). Similarly, high agreement of variant calling was observed when compared with the truth set validated by ddPCR, especially under the SNVer and VarScan2 tools (Figure 1C). For TargetSeq One panel, SNVer and VarScan2 exhibited values close to 100% in the F-score, recall, and precision of SNP&InDel (Supplementary Figure S2) under the almost equal sequencing depth (Supplementary Table S5). For panel sequencing of six breast cancer and ovarian cell lines samples, all five tools performed flawlessly in three samples, while, in the remaining three samples, the detection results of SNVer and VarScan2 were closest to the true set (Supplementary Table S6).

Pearson correlation coefficient (*r*) heatmap (Figure 1D) revealed that under the same software, similar overall performance was presented across-platforms, with slightly higher consistency from SiNVICT with average *r*: 0.86 (0.68–0.97), SNVer with 0.86 (0.71–0.97), and VarScan2 with 0.90 (0.75–0.98) approaches. Intriguingly, data from SiNVICT and SNVer pipelines showed high correlation across all 50 datasets (average *r*: 0.84). Less accordance was observed in data from five pipelines under the same platform, especially for HC (average *r*: 0.71) and Mutect2 (average *r*: 0.78), consistent with the variant calling results (Figures 1A–C). In addition, the data of three technical replicates on the same platform indicated high correlation except for a few outliers, such as FS_HC samples. Hereafter, we combined three replicates to investigate the detection number of high-confidence variants among tools and platforms (Figure 1E). Up to 93.4% (198/212) concordance was obtained from all datasets, of which, the FS platform performed better with more unique mutations, particularly under the SNVer and VarScan2 callers, reproducing the above-described results.

Overall, the results of SNP&InDel calling suggested that software may have a greater impact on variant calling than platform for the target sequencing datasets, and FS performed better under SNVer and VarScan2 approaches.

### 3.3 Assessment of variant calling at different sequencing depths

Sequencing to greater depths increases the reproducibility of variant detection but at the expense of longer turnaround times, increased computational complexity, and greater cost. Focusing on the best-performed FS platform and the SNVer and VarScan2 pipelines, we compared the differences in variant calling time, memory usage, SNP_recall and InDel_recall with radar chart at different sequencing depths, expecting to recommend a balance between sequencing depth and variant detection sensitivity (Figures 1F,G). As expected, the calling time and memory usage progressively increased with increasing depth of coverage, from 250× to 2000×. Additionally, we saw no apparent improvement in SNP&InDel recall percentage with increasing depth from 500×. At the given depth of 250×, the SNVer pipeline identified a median recall of 99.48% for SNPs and 99.44% for InDels compared with a recall of 98.96% for SNPs and 100% for InDels using the VarScan2 pipeline. It suggests that the lowest depth to discover all high-confidence mutations within the truth set is 500× for the FS datasets analyzed by the SNVer or VarScan2 pipeline. Concerning turnaround time and memory usage, the VarScan2 performed better than SNVer tool.

### 3.4 Concordance of annotated genes from different datasets

To further explore the effects of gene annotation on different platforms and software tools, we compared the concordance of the annotated genes based on their rVAF. Supplementary Figure S3 showed the visual difference between the VAF of all datasets and truth set, and white represents no difference. The heatmap returned highly concordant results for datasets analyzed by different pipelines, while some perceptible differences can be noticed when using SNVer and VarScan2 callers. Specifically, noticeable increases in rVAF were observed by VarScan2, conversely, obvious decreases for the same genes were detected by SNVer. Focusing on variants with low mutation frequency (*MET*, *KIT* and *NRAS* genes), we were delighted to find that SiNVICT, SNVer and VarScan2 approaches were friendly to all platforms with high consistency, including the novel FS platform. HC caller performed worst across different platforms with more notable dissimilarity. Notably, for *EGFR* mutation with very low VAF (4.5%), five platforms failed to discern it under HC and Mutect2 pipelines. The absolute VAF (Supplementary Tables S7, S8) also indicated high concordance for datasets compared to the truth set.

### 3.5 Benchmarking somatic mutations and TMB distributing in the complete panel

Furthermore, we broadened to the global patterns of somatic variations observed in the panel range to obtain comprehensive insight. The common germline variants and benign variants were filtered out when VAF >0.1%. Detailed information on the somatic mutation spectrum in each dataset is represented as a waterfall plot (Supplementary Figure S4). Overall, the driver somatic variants, including missense, stop-gained, frame-shift, and splice mutations, showed very high agreement among 50 datasets from different platforms and pipelines. Nevertheless, HC pipeline exhibited more discordance, such as genes *NSD1, BRAF*, *STAT3*, and *GRM3*. With respect to TMB, all sequencers and pipelines returned comparable concordance, with higher TMB number when using the SiNVICT and VarScan2 tools, which may be explained by the higher number of the somatic mutations (Supplementary Figure S5). The number of somatic mutations from three technical replicates indicated that the highest shared-variants for FS platform were obtained fromVarScan2 (93.67%), for GL platform from SiNVICT (92.33%), for NA platform from SiNVICT (82.30%). In contrast, HC shared inferior proportion of common variants for the FS (42.50%), consistent with the results from waterfall plot. While Mutect2 performed worst for GL (55.53%) and NA (28.13%) platforms. Need to note, we detected a novel set of somatic variant sitesandgenes outside the truth set published by HD832 through the intersection of our 50 datasets (Table 1). These high-confidence variants were expected to be a supplement to the published truth set of HD832, offering new insights into the tumor variant research.

**TABLE 1:** Novel somtaic variant genes identified in our study that are not included in the truth set published by HD832.

GRCh37 (hg19)		Ref	Alt	Gene	Variant type	Cosmic ID (v97)	Amino acid	Sequence ontology term	Average allele frequency (%)	Depth (×)
Chr	Location									
chr1	36,933,199	T	C	*CSF3R*	SNP	COSV58970806	p.Thr640Ala	missense_variant	29.05	809.66
chr2	99,154,426	G	A	*INPP4A*	SNP	COSV50811933	p.Val190Ile	missense_variant	30.35	730.08
chr2	202,137,387	G	A	*CASP8*	SNP	COSV51857659	p.Met205Ile	missense_variant	30.56	532.12
chr3	49,936,300	C	A	*MST1R*	SNP	COSV56572804	p.Gln516His	missense_variantandsplice_region_variant	31.82	538.80
chr3	187,446,153	C	T	*BCL6*	SNP	COSV51656257	p.Ser512Asn	missense_variant	30.31	658.08
chr5	39,002,766	A	G	*RICTOR*	SNP		p.Leu88Ser	missense_variantandsplice_region_variant	13.74	346.00
chr6	111,983,012	T	C	*FYN*	SNP	COSV57622174	p.Tyr515Cys	missense_variant	28.68	723.56
chr7	50,444,272	G	A	*IKZF1*	SNP	COSV58792485	p.Gly68Arg	missense_variant	21.94	708.74
chr8	93,017,471	G	GT	*RUNX1T1*	InDel		p.Gln264fs	frameshift_variant	13.71	780.74
chr10	90,767,575	A	T	*FAS*	SNP	COSV58239879	p.Arg105Ser	missense_variant	23.66	575.32
chr12	6,709,179	C	A	*CHD4*	SNP			splice_acceptor_variantandintron_variant	11.67	511.06
chr13	102,375,201	T	C	*FGF14*	SNP	COSV65936716	p.Asn247Asp	missense_variant	8.62	609.44
chr15	45,003,808	C	T	*B2M*	SNP	COSV62564195	p.Gln22*	stop_gained	30.25	813.56
chr15	45,008,529	C	T	*MIR10393*	SNP	COSV62562883	p.Arg117*	stop_gainedandsplice_region_variant	10.32	360.76
chr15	66,782,085	A	G	*MAP2K1*	SNP	COSV57235242, COSV57235143	p.Asp351Gly	missense_variant	31.95	528.86
chr15	73,994,743	T	C	*CD276*	SNP	COSV59205301	p.Val76Ala	missense_variant	31.09	767.76
chr15	93,480,818	C	CA	*CHD2*	InDel		p.Val175fs	frameshift_variant	20.62	562.08
chr17	30,310,119	G	T	*SUZ12*	SNP		p.Gly340Val	missense_variant	31.03	449.58
chr19	36,214,360	G	T	*KMT2B*	SNP	COSV55867628	p.Cys1005Phe	missense_variant	29.18	552.34
chr19	50,906,813	T	C	*POLD1*	SNP		p.Phe401Leu	missense_variant	11.40	747.88
chr20	31,379,499	G	A	*DNMT3B*	SNP	COSV52422156	p.Met302Ile	missense_variant	23.90	593.92
chr20	3,9,751,901	G	T	*TOP1*	SNP		p.Trp754Cys	missense_variant	28.17	668.46

Note: These variants were not recorded in both ClinVar and GnomAD., Chr: Chromosome, Ref: Reference, Alt: Alteration.

Gene names are listed in italics.

## 4 Discussion

NGS-based targeted sequencing has gained prominence in assessing genetic alterations in cancer, influencing the ongoing development of personalized medicine. Compared to WGS and WES, TS could minimise manpower, running time, sequencing data, storage space and computational demand by merely probing into the interesting targets, thus being economical and cost-effective (Bewicke-Copley et al., 2019). The excellent coverage depth is the icing on the cake for its sensitivity and specificity. Whereas, an insufficient clinical consensus on sequencing platforms and variant calling tools confuses researchers and clinicians. We evaluated five variant calling tools on four sequencing platforms to identify the possibilities and limits of variant screening.

Turn-around-time and sensitivity are foundational to good clinical diagnosis and essential to achieving optimal patient outcomes. Specifically, early and reliable tests of genetic biomarkers available in patients may decrease the use of more invasive testing, under- and overtreatment, and reduces the adverse events associated with inappropriately targeted therapies (Singh et al., 2019; Wurcel et al., 2019). In this study, we observed high concordance of mutation calling across different sequencers and software tools generally, albeit with some outlier values and minor differences (especially for HC and Mutect2 pipelines). Of note, the FS platform showed higher sensitivity (100%) in high-confidence variants calling than the other three platforms, when analyzed by SNVer and VarScan 2 software algorithms (Figures 1A–C). Considering the dissimilarity of variant calls across different bioinformatics tools for datasets from the same sequencer (Figures 1D,E), we appraised that software appears to contribute greater to the variant detection. In addition, comparing the sequencing time of four platforms in this study, the FS platform shows dominance with the shortest sequencing time (FS, ∼21 h; NA, ∼25 h; NS, ∼26 h; GM, ∼43 h) at the sequencing mode PE150.

Due to the high-depth nature of TS, special attention needs to be taken to the relationship between the sequencing depth and precision/sensitivity of mutation detection. However, there is no consensus on the minimum required depth in a clinical setting for TS. Previous studies attempted to leverage the power of Illumina technologies to recommend an appropriate depth for TS. Nevertheless, there is still no consistent standard presently, ranging from 100× to 1,000× (Cortini et al., 2017; Bewicke-Copley et al., 2019; Koboldt, 2020). Further studies are thus needed to define the proper sequencing depth, especially for novel platforms. For datasets sequenced on the FS platform and called by SNVer and VarScan 2 algorithms, the sufficient sequencing depth for the TSO500 panel was 500×, which enables 100% detection of high-confidence variants (Figures 1F,G).

Accurate variant calling in NGS data is well known to have a critical impact on the downstream analysis and interpretation processes, for which, a proper variant detection algorithm is important for high sensitivity and specificity (Sandmann et al., 2017). Through the benchmarking analysis of five popular variant calling tools, we noted that the choice of software algorithm represented an important factor for variant detection. For example, SiNVICT, SNVer and VarScan 2 had high tool-specific “unique” calls when compared with the truth set (Figure 1E), while HC seemed to have the fewest concordant calls (Figures 1D,E). However, focusing on the VAF of the annotated genes, we noticed some perceptible differences between the truth set and the values analyzed by SNVer and VarScan2 callers (Supplementary Figure S3). These findings indicated that multiple analysis algorithms should be used to obtain a consensus to reduce false-discovery when diagnosing cancer-associated mutations.

While we provide a useful benchmark for the researcher to choose sequencing platforms and analysis pipelines, there are some limitations. Because of time and budget constraints, we only analyzed two standard cancer samples (OncoSpan FFPE (HD832) and Twist cfDNA Pan-cancer Reference Standard) with five pipelines. Although these samples provide reliable variant information, FFPE-based positive and negative patient samples would generate more complete evaluations. To acquire a comprehensive profile of sequencing platforms and pipelines, multiple samples from the HD832 cell line and clinical cancer FFPE samples should be enrolled for future research to enable ultimate clinical applications of the sequencers and variant callers.

## 5 Conclusion

There was good concordance of variant calling across different platforms and pipelines. FS platform showed highest sensitivity (100%) in high-confidence variants calling when analyzed by SNVer and VarScan 2 algorithms. Our study provides a standardized target sequencing resource to benchmark new bioinformatics protocols and sequencing platforms. Moreover, the variant calling performances of five software on three typical tumor samples provide a valuable reference for future tumor variant research.

## Data Availability

The datasets presented in this study can be found in online repositories. The names of the repository/repositories and accession number(s) can be found below: https://db.cngb.org/search/project/CNP0004275/, CNP0004275.
