# Supplementary material for: Systematic comparison of variant calling pipelines of target genome sequencing cross multiple next-generation sequencers
Source: Front Genet. 2024 Jan 4;14:1293974. doi: 10.3389/fgene.2023.1293974 (PMC10794554; doi:10.3389/fgene.2023.1293974)
Supplement: Supplementary file 3 [file Presentation1.PDF]

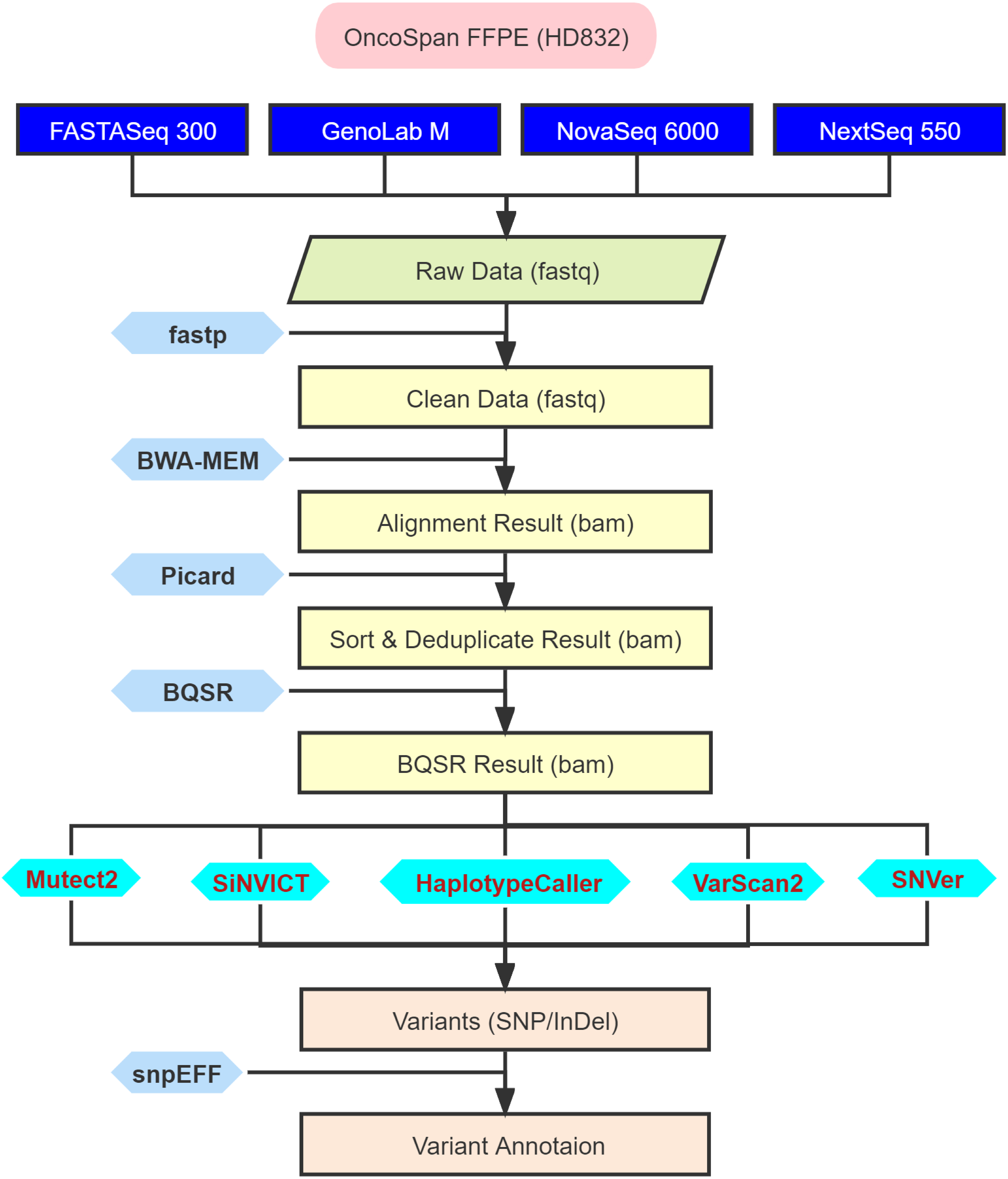

OncoSpan FFPE (HD832)
FASTASeq 300
GenoLab M
NovaSeq 6000
NextSeq 550
Raw Data (fastq)
fastp
Clean Data (fastq)
BWA-MEM
Alignment Result (bam)
Picard
Sort & Deduplicate Result (bam)
BQSR
BQSR Result (bam)
Mutect2
SiNVICT
HaplotypeCaller
VarScan2
SNVer
Variants (SNP/InDel)
snpEFF
Variant Annotaion
