# Supplementary material for: Systematic comparison of variant calling pipelines of target genome sequencing cross multiple next-generation sequencers
Source: Front Genet. 2024 Jan 4;14:1293974. doi: 10.3389/fgene.2023.1293974 (PMC10794554; doi:10.3389/fgene.2023.1293974)
Supplement: Supplementary file 4 [file Presentation4.PDF]

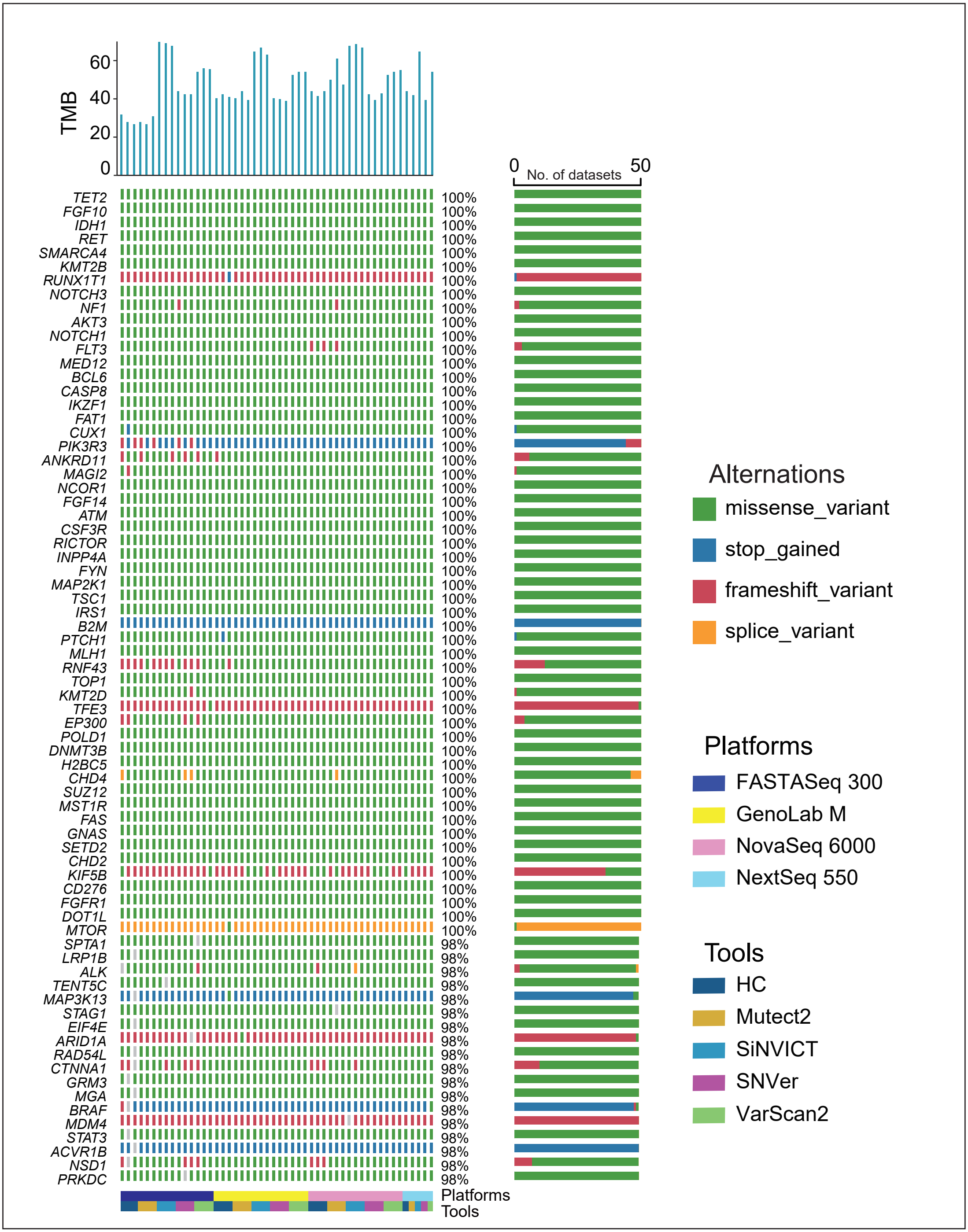
TMB
60
40
20
0
0
No. of datasets
50
TET2
FGF10
IDH1
RET
SMARCA4
KMT2B
RUNX1T1
NOTCH3
NF1
AKT3
NOTCH1
FLT3
MED12
BCL6
CASP8
IKZF1
FAT1
CUX1
PIK3R3
ANKRD11
MAGI2
NCOR1
FGF14
ATM
CSF3R
RICTOR
INPP4A
FYN
MAP2K1
TSC1
IRS1
B2M
PTCH1
MLH1
RNF43
TOP1
KMT2D
TFE3
EP300
POLD1
DNMT3B
H2BC5
CHD4
SUZ12
MST1R
FAS
GNAS
SETD2
CHD2
KIF5B
CD276
FGFR1
DOT1L
MTOR
SPTA1
LRP1B
ALK
TENT5C
MAP3K13
STAG1
EIF4E
ARID1A
RAD54L
CTNNA1
GRM3
MGA
BRAF
MDM4
STAT3
ACVR1B
NSD1
PRKDC
100%
98%
Platforms
Tools
Alternations
missense_variant
stop_gained
frameshift_variant
splice_variant
Platforms
FASTASeq 300
GenoLab M
NovaSeq 6000
NextSeq 550
Tools
HC
Mutect2
SiNVICT
SNVer
VarScan2
