# Supplementary figures and images for: Systematic comparison of variant calling pipelines of target genome sequencing cross multiple next-generation sequencers
Source: Front Genet. 2024 Jan 4;14:1293974. doi: 10.3389/fgene.2023.1293974 (PMC10794554; doi:10.3389/fgene.2023.1293974)

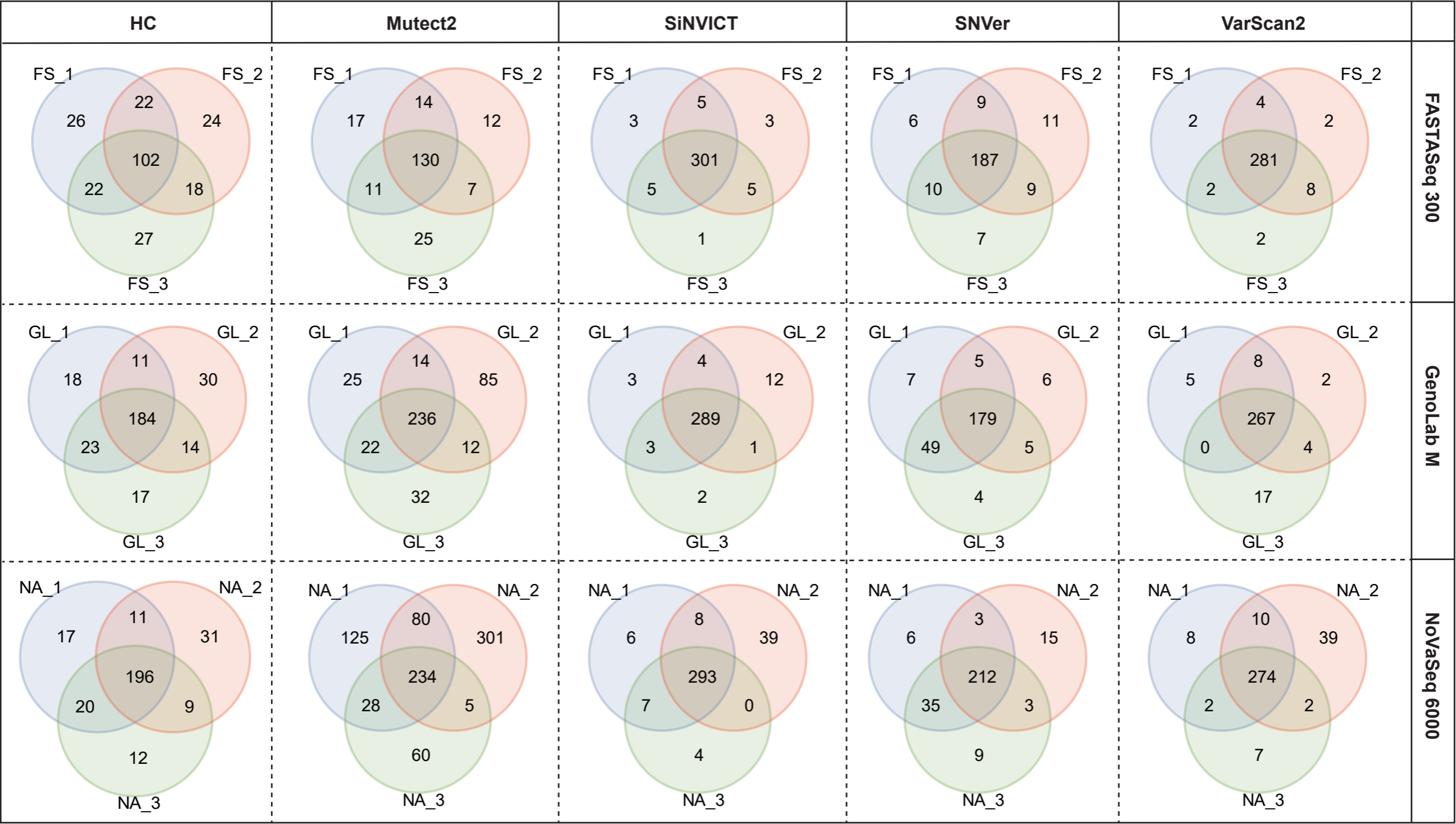

Supplement: Supplementary file 5 [file Presentation5.PDF]

a

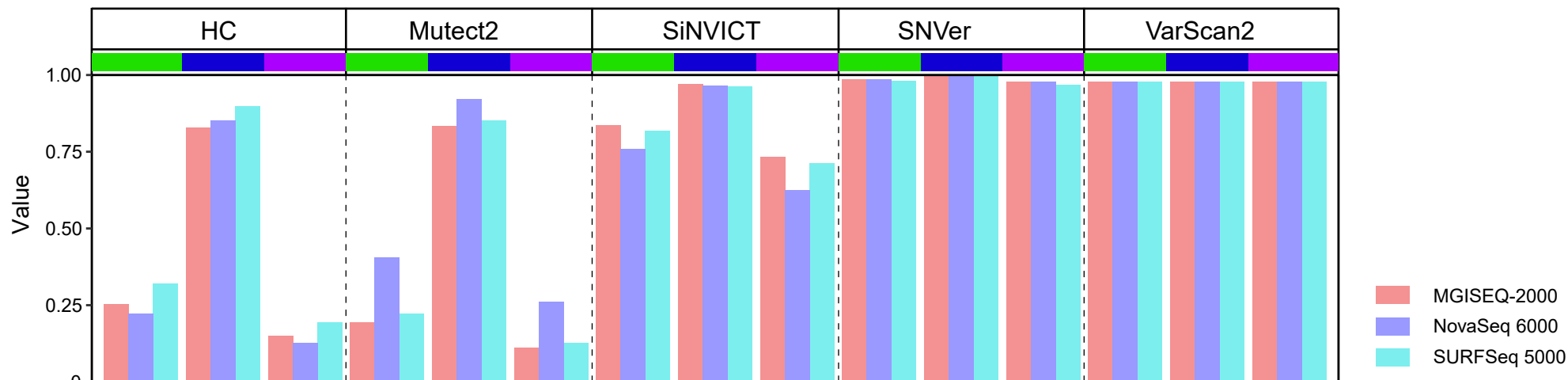

b

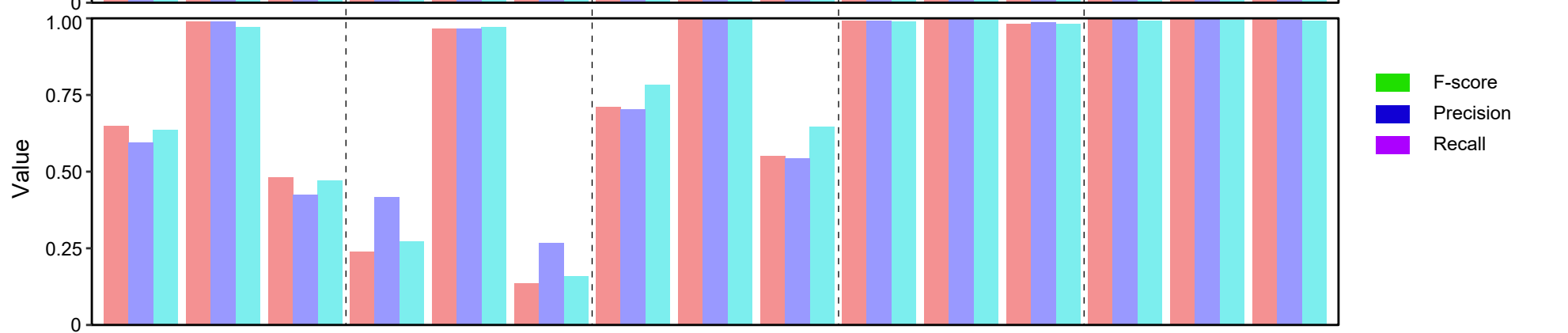

Supplement: Supplementary file 9 [file Presentation2.PDF]

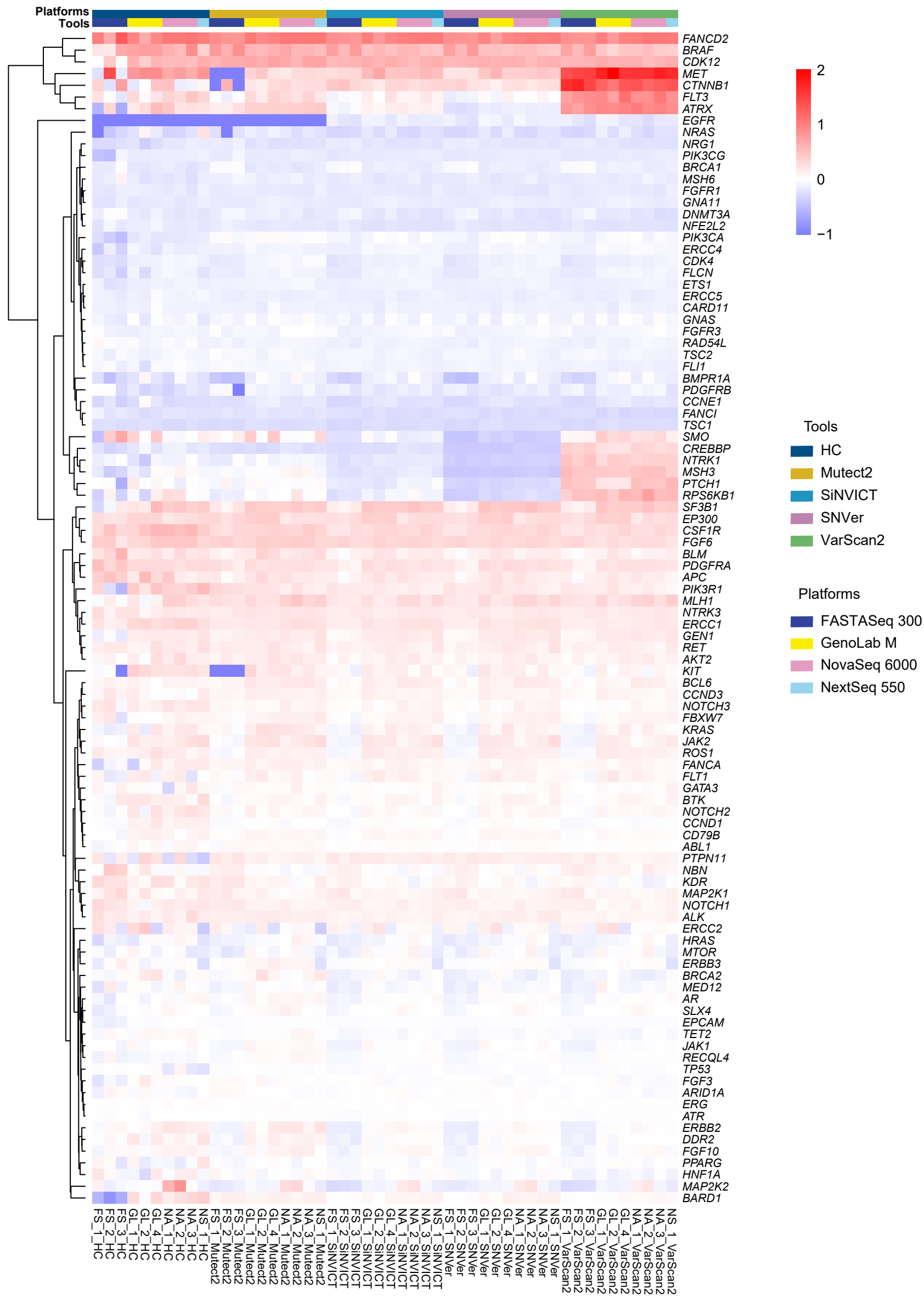

Supplement: Supplementary file 13 [file Presentation3.PDF]
