# Supplementary material for: Systematic comparison of variant calling pipelines of target genome sequencing cross multiple next-generation sequencers
Source: Front Genet. 2024 Jan 4;14:1293974. doi: 10.3389/fgene.2023.1293974 (PMC10794554; doi:10.3389/fgene.2023.1293974)
Supplement: Supplementary file 14 [file DataSheet1.docx]

Supplementary Material

# Supplementary Figures and Tables


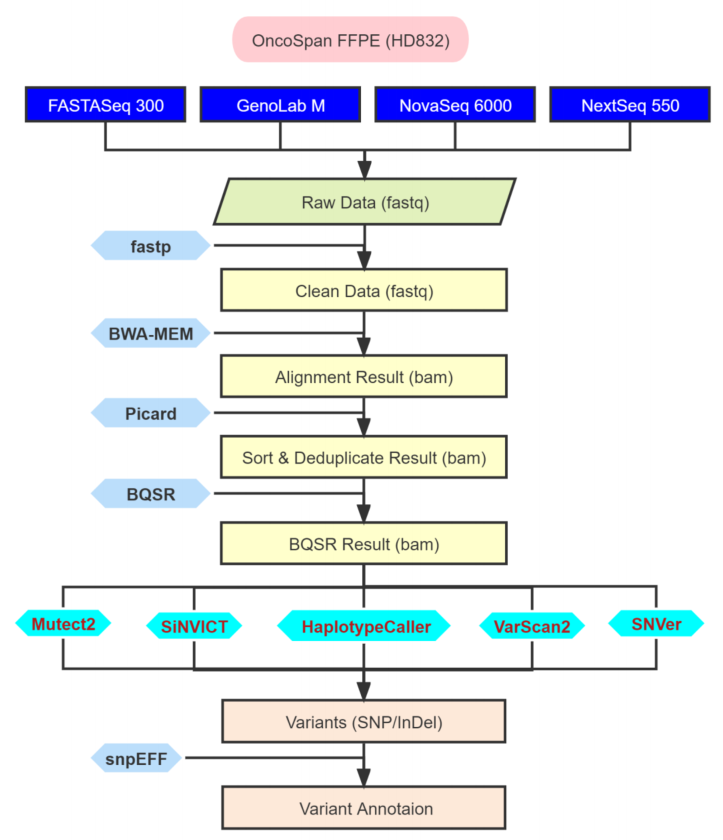


**Figure S1**. The flowchart of variant calling across four sequencing platforms and five pipelines. Squares in the flowchart represent data files, and hexagon indicate software. HC, GATK_HaplotypeCaller; Mutect2, GATK_Mutect2.


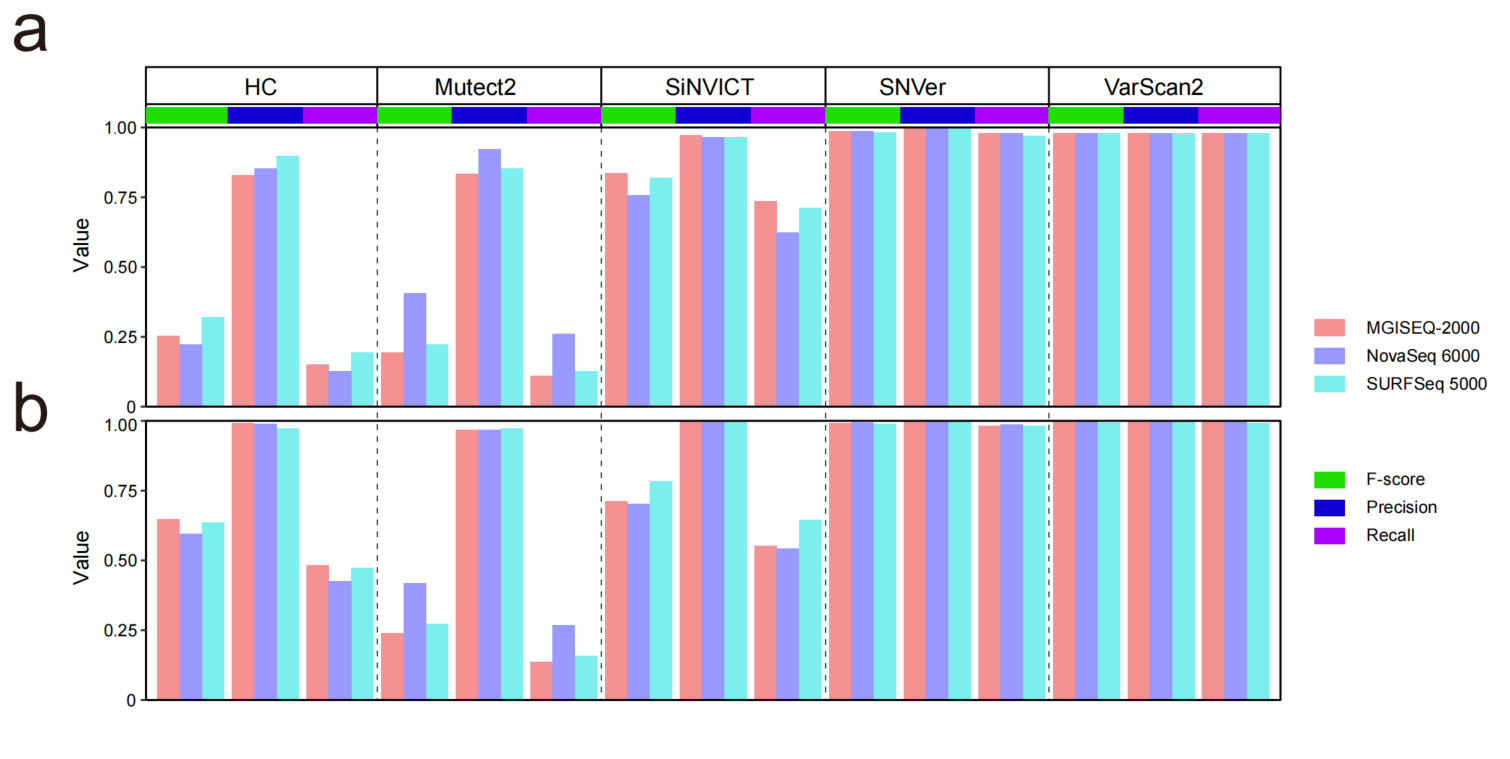


**Figure S2.** Benchmarking analyses of variants calling performances compared with the truth set by TargetSeq One kit enriched. (a) SNP and (b) InDel. HC, GATK_HaplotypeCaller; Mutect2, GATK_Mutect2.


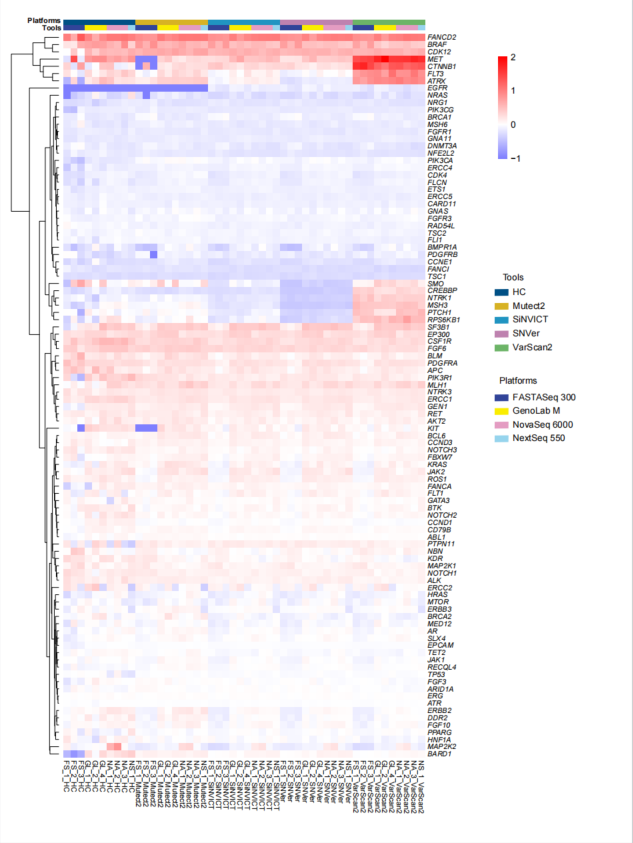


**Figure S3**. Concordance of relative frequency of annotated variant genes from 50 datasets.

The scale represent difference between the VAF of each dataset and the reference VAF. The calculation formula is [(VAF of the dataset) - (truth VAF)] / (truth VAF). The colourful bars at the top indicates different tools and platforms. FS, FASTASeq 300; NA, NovaSeq 6000; NS, NextSeq 550; GL, GenoLab M; HC, GATK_HaplotypeCaller; Mutect2, GATK_Mutect2.


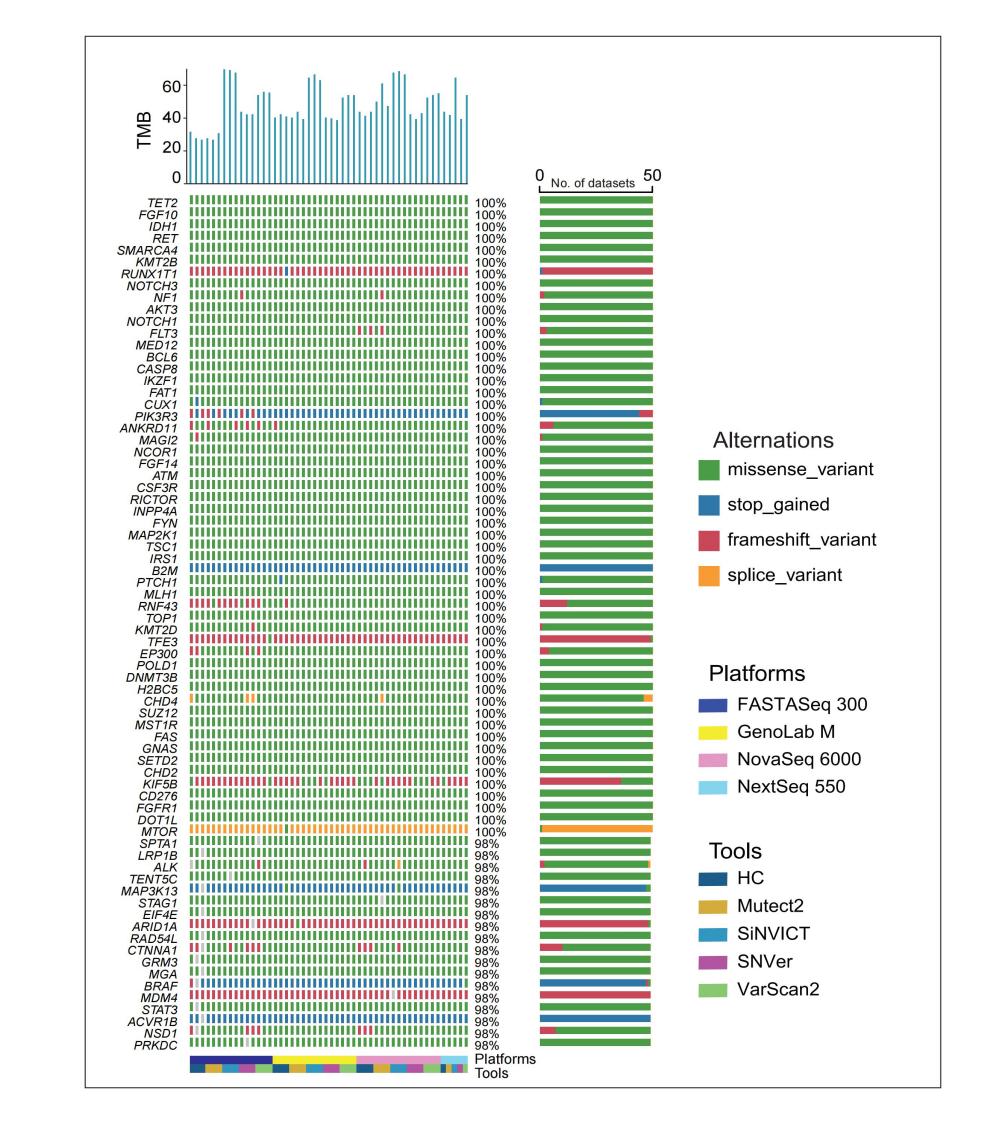


**Figure S4**. Landscape of somatic mutation profiles for 50 datasets within the panel. Each column corresponds to a single dataset and the colors are indicative of the type of mutation. The barplot at the top panel shows the number of TMB. The waterfall plot depicts the somatic mutation information of each gene. The vertical plot on the right represents the ratio of mutations in each gene, color coded by driver somatic mutation types. HC, GATK_HaplotypeCaller; Mutect2, GATK_Mutect2.


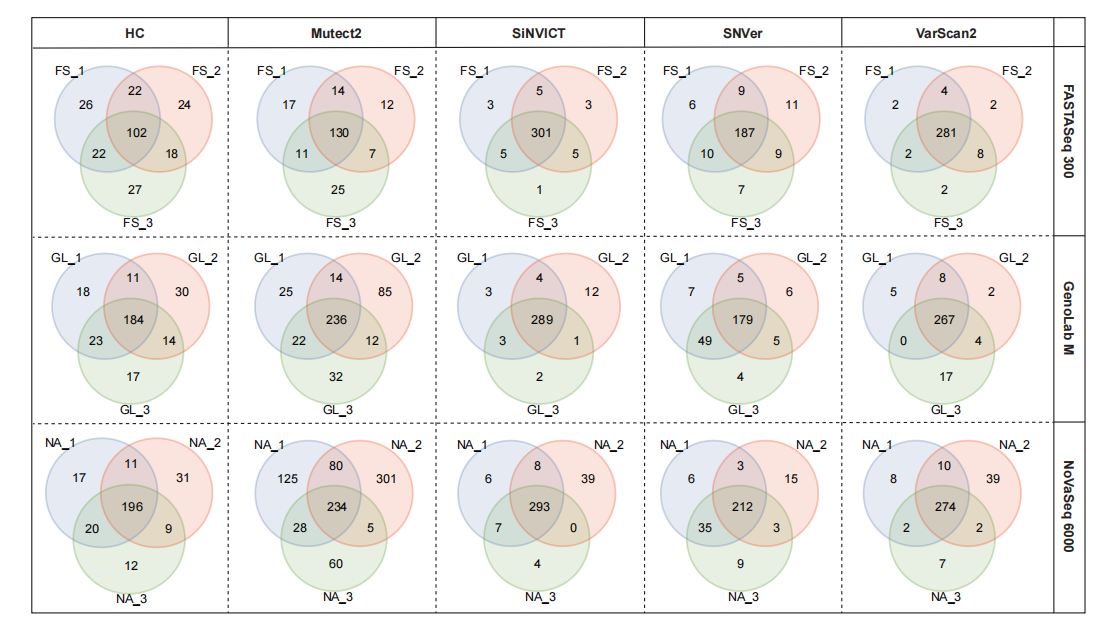


**Figure S5**. Venn diagrams represent the overlap somatic variants in three technical replicates called by five tools from FS, GL and NA platform. FS, FASTASeq 300; GL, GenoLab M; NA, NovaSeq 6000; HC, GATK_HaplotypeCaller; Mutect2, GATK_Mutect2.

**Table S1.** The character of the tools used in the study

This table is a large datasets, so we have uploaded it as additional files.

**Table S2.** Summary of the sequencing datasets.

|  | **FS_1** | **FS_2** | **FS_3** | **NA_1** | **NA_2** | **NA_3** | **NS_1** | **GL_1** | **GL_2** | **GL_3** |
| --- | --- | --- | --- | --- | --- | --- | --- | --- | --- | --- |
| **Reads (M)** | 69.15 | 64.13 | 65.20 | 68.06 | 60.80 | 71.05 | 69.09 | 67.89 | 61.02 | 65.80 |
| **Bases (G)** | 10.37 | 9.62 | 9.78 | 10.21 | 9.12 | 10.66 | 10.23 | 10.18 | 9.15 | 9.87 |
| **Q20 (%)** | 96.46 | 96.45 | 96.57 | 97.59 | 96.43 | 98.18 | 94.05 | 97.40 | 96.83 | 96.81 |
| **Q30 (%)** | 90.79 | 90.57 | 91.05 | 92.69 | 89.95 | 94.13 | 87.88 | 91.30 | 91.37 | 89.61 |
| **GC (%)** | 47.93 | 47.91 | 47.90 | 50.42 | 50.28 | 50.36 | 49.00 | 47.15 | 48.97 | 46.17 |
| **Mapped Data (%)** | 99.66 | 99.67 | 99.67 | 100.00 | 100.00 | 100.00 | 100.00 | 99.98 | 99.63 | 99.99 |
| **duplicate reads (%)** | 72.61 | 72.95 | 72.94 | 64.18 | 58.77 | 59.50 | 55.70 | 54.23 | 54.09 | 54.84 |
| **Average depth (rmdup)** | 2556 | 2374 | 2411 | 3012 | 2690 | 3138 | 3042 | 3015 | 2688 | 2920 |
| **covered > 0x (%)** | 99.70 | 99.70 | 99.70 | 99.32 | 99.27 | 99.36 | 99.26 | 99.18 | 99.42 | 99.14 |
| **covered >= 4x (%)** | 99.66 | 99.67 | 99.66 | 99.15 | 99.09 | 99.16 | 99.05 | 98.97 | 99.21 | 98.89 |
| **covered >= 10x (%)** | 99.60 | 99.61 | 99.61 | 98.99 | 98.91 | 99.01 | 98.79 | 98.75 | 98.99 | 98.66 |
| **covered >= 30x (%)** | 99.46 | 99.43 | 99.43 | 98.73 | 98.63 | 98.79 | 98.42 | 98.45 | 98.65 | 98.30 |
| **covered >= 100x (%)** | 98.94 | 98.87 | 98.90 | 98.29 | 98.11 | 98.37 | 97.72 | 97.84 | 98.14 | 97.63 |

FS, FASTASeq 300; NA, NovaSeq 6000; NS, NextSeq 550; GL, GenoLab M

**Table S3** The average depth for 131 genes enriched by TSO500 panel

This table is a large datasets, so we have uploaded it as additional files.

**Table S4** The variants calling performances among sequencers and tools

This table is a large datasets, so we have uploaded it as additional files.

**Table S5**. Summary of Twist cfDNA Pan-cancer Reference Standard sequencing datasets by TargetSeq One kit enriched.

| **Platfrom** | **Bases (Gb)** | **Q20(%)** | **Q30(%)** | **GC(%)** | **Mapped Data (%)** | **duplicate reads (%)** | **Average depth (rmdup)** | **covered > 0x (%)** | **covered >= 4x (%)** | **covered >= 10x (%)** | **covered >= 30x (%)** | **covered >= 100x (%)** |
| --- | --- | --- | --- | --- | --- | --- | --- | --- | --- | --- | --- | --- |
| SURFSeq 5000 | 4.79 | 97.09 | 93.24 | 48.90 | 99.44 | 57.34 | 1193 | 99.69 | 99.47 | 99.19 | 98.62 | 97.49 |
| MGISEQ-2000 | 4.72 | 97.34 | 92.66 | 48.40 | 99.83 | 32.68 | 1091.96 | 99.60 | 99.24 | 98.91 | 98.25 | 97.32 |
| NovaSeq 6000 | 4.84 | 98.20 | 94.99 | 49.89 | 99.70 | 58.93 | 1182.99 | 99.70 | 99.45 | 99.18 | 98.68 | 97.59 |

**Table S6**. The SNP and InDel calling results of cancer cell lines

This table is a large datasets, so we have uploaded it as additional files.

**Table S7** The variant allele frequency of the annotated genes for TSO500.

This table is a large datasets, so we have uploaded it as additional files.

**Table S8** The variant allele frequency of the annotated genes for Twist cfDNA Pan-cancer Reference Standard by TargetSeq One kit enriched.

This table is a large datasets, so we have uploaded it as additional files.
